# Imperforate Hymen Presenting as Acute Urinary Retention in A 14-Year-Old Nigerian Girl

**DOI:** 10.4103/2006-8808.73623

**Published:** 2010

**Authors:** Obi O. Anselm, Uzor H. Ezegwui

**Affiliations:** *Urology Unit, Department of Surgery, Federal Medical Centre, Abakaliki, Nigeria*; 1 *Department of Obstetrics and Gynaecology, Federal Medical Centre, Abakaliki, Nigeria*

**Keywords:** Acute urinary retention, imperforate hymen, haematocolpos

## Abstract

Acute urinary retention in adolescent females is rare, just like imperforate hymen. We present a case of acute urinary retention secondary to imperforate hymen in a 14-year-old Nigerian girl. Its diagnosis and treatment are discussed with a brief review of literature. We highlight the need for a thorough evaluation in the female patient presenting with acute urinary retention, and also the need to provide better health facilities in rural areas in developing countries such as ours.

## INTRODUCTION

While acute urinary retention is quite common in men, it is a rare occurrence in females. This is because of their short urethra and peculiar anatomic relationships, and as such it is rarely plagued by obstructing lesion.[1] When it does occur it is usually due to an obstructing pelvic or perineal mass. One of such obstructing lesions is a colpomenorrhoea, which is the accumulation of menstrual blood above an imperforate hymen leading subsequently to the distention of the vagina to a variable degree. The distention of the vagina leads to stretching of the urethra, which is an integral part of the anterior vaginal wall and eventually urinary retention. A case of imperforate hymen that presented with acute urinary retention is described.

## CASE REPORT

**Figure 1:**
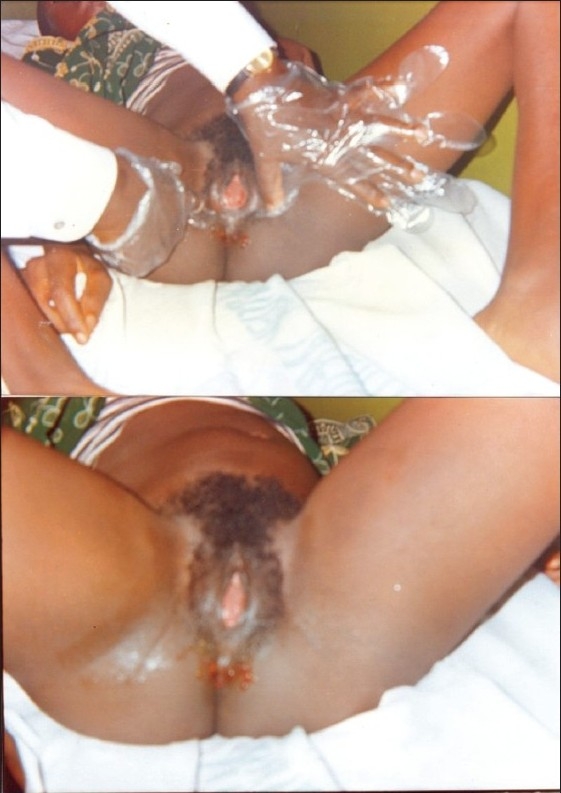
Nigerian girl, 14-year-old, with acute urinary retention and imperforate hymen

**Figure 2:**
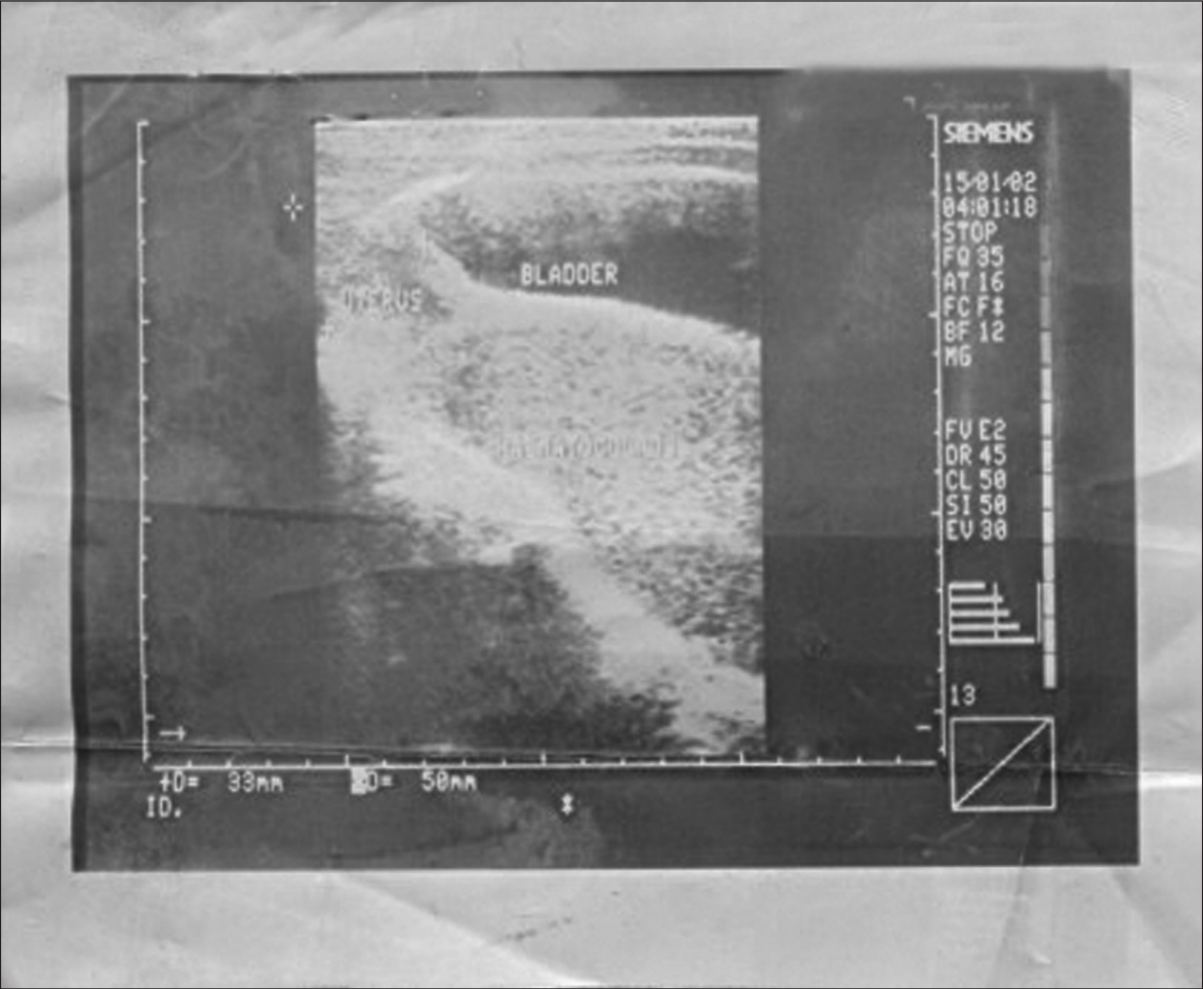
Ultrasound scan of the patient showing hematocolpos

We present the case of a 14-year-old Nigerian girl who presented to our urology service with acute urinary retention. Two weeks prior to presentation she had been experiencing increasing difficulty in passing urine, associated with lower abdominal pain, but no fever or dysuria was observed. She had experienced two episodes of urinary retention, which were relieved by catheterization in a rural health centre. Prior to this she had been experiencing cyclical abdominal pain for about 10 months but had never menstruated. Physical examination revealed a young girl in painful distress. She was not pale or febrile to touch. The significant findings were in the abdomen and external genitalia: She had a firm tender suprapubic swelling that was about 20 weeks size. One could get above the swelling but not below it. Vaginal examination revealed normal vulva with an unobstructed external urethral meatus, but a bulging imperforate hymen with a bluish discolouration [Figure 1]. Bimanual examination through the rectum also revealed a markedly distended vagina bulging into the anterior rectal wall. A diagnosis of acute urinary retention secondary to hematocolpos was made and this was later confirmed by abdominal ultrasound, which showed gross distention of the vagina with heteroechoic fluid collection [Figure 2]. The uterus and adnexae and the rest of the abdominal viscera were normal. The initial management consisted of aseptic catheterization with a size 16f foley urethral catheter to relieve the acute urinary retention. Amber colored urine (750 ml) was drained. The catheter was left indwelling and spigotted. Urine analysis, culture, and sensitivity was normal. Her hemoglobin was 11 g/dl. Subsequent management consisted of hymenotomy at the next operating day; a cruciate incision was made on the imperforate hymen and approximately 1L of viscous chocolate-colored altered blood drained passively. The urethral catheter was removed and the patient was discharged home the next day. At the one and three months follow-up she was completely symptom free and had normal ultrasound findings. She had a 29 day menstrual cycle and 4 days of menstruation.

## DISCUSSION

Untreated imperforate hymen is a rare but recognized cause of acute urinary retention.[2–4] The hymen appears as a thin but discernible tissue plate by the 3^rd^ month of intrauterine life separating the vagina from the urogenital sinus. It usually develops a small opening during perinatal life. Failure to do so results in an imperforate hymen.[5] Some cases are recognized at birth because of mucocolpus accumulation[6] but most girls present the symptom at puberty.[4,7] This young girl presented when she was aged 14 years. The severity ranges from isolated imperforate hymen to complete vaginal atresia with skeletal and urinary abnormalities. This makes ultrasound scanning of the kidneys and ureters necessary when imperforate hymen is suspected.

The symptoms arise mainly from accumulation of menstrual blood flow into the vagina (Hematocolpos) and uterus (Hematometra) and may cause relatively little discomfort. If left untreated, distension of the vagina will lead to stretching and obstruction of the urethra because of its very close anatomic relationship with the anterior vaginal wall, as was the case in this index patient. The management is to ensure drainage of the vagina and uterus by means of a cruciate incision of the imperforate hymen under aseptic conditions.[8,9] The uterus should not be squeezed in the course of the drainage because this may cause the altered menstrual blood to flow back through the fallopian tubes into the peritoneal cavity with the possibility of tubal adhesions and endometriosis, both of which may lead to infertility.[6]

The diagnosis and treatment is easy but majority of patients are rural dwellers with little or no access to health facilities. Patients have to travel long distances to obtain quality medical services just like the index patient.

## CONCLUSION

Untreated imperforate hymen can result in acute urinary retention when the vagina and uterus are sufficiently distended by accumulated menstrual blood. Because urinary retention is rare in female patients, there is need for a thorough examination including ultrasound scan to determine its exact cause. Finally, there is need for health education of rural dwellers in addition to the provision of adequate health facilities in the rural areas.
